# Pregnant Mothers Diversified Dietary Intake and Associated Factors in Southwest Ethiopia: A Cross-Sectional Study

**DOI:** 10.1155/2022/4613165

**Published:** 2022-08-02

**Authors:** Yihun Tariku, Kaleab Baye

**Affiliations:** ^1^Department of Public Health, Arba Minch College of Health Sciences, Arba Minch, Ethiopia; ^2^Center for Food Science and Nutrition, Addis Ababa University, Addis Ababa, Ethiopia

## Abstract

**Background:**

Dietary diversity is very critical for fetal growth and development, as well as for the health and wellbeing of the mother. In Ethiopia, 41% of pregnant mothers consume diversified diets. There has been no study assessing whether pregnant women in southwest Ethiopia consume a varied diet.

**Objective:**

To estimate the proportion of adequate diversified dietary intake and to determine whether there is an association between diversified dietary intake and mothers' social capital.

**Design:**

A community-based cross-sectional study was conducted on 367 pregnant women in South West Ethiopia. A 24-hour multiple-pass dietary recall method was used to measure dietary intake and the 10 food groups of FANTA/FAO 2016 were used to analyze dietary diversity. Social capital was assessed based on the number of social networks in which a mother participated.

**Result:**

The proportion of diversified dietary intake was 14.7% (95% CI: 11.1, 18.3). Pregnant mothers who had social capital, had their own income, had emotional support from their husbands, were from a rich family, and had a frequent dietary intake were statistically associated with a diversified dietary intake, with an AOR of 7.8 (95% CI 1.02, 2.3 (95% CI 1.12, 4.44), 4.0, (95% CI 1.16, 13.7), 59.19), 2.3 (95% CI 1.04, 5.26), and 1.5 (95% CI 1.04, 2.07), respectively.

**Conclusion:**

Diversified dietary intake was found to be lower than previously reported, and it was associated with social capital. Methodologically rigorous studies are required to verify the association between social capital and adequate diversified dietary intake.

## 1. Introduction

An inadequately diversified diet during pregnancy worsens maternal health and causes adverse birth outcomes. In Ethiopia, a low proportion (41%) of pregnant mothers obtained an adequately diversified diet, and as a result, many children are suffering from undernutrition [1]. According to the 2016 Ethiopian Demographic and Health Survey (EDHS) report, 13% of births were low birth weight (Central Statistical Agency [2]. The most recent EDHS revealed that 37%, 21%, and 7% of children under 5 years of age were stunted, underweight, and wasted, respectively (Ethiopian Public Health Institute [3]. Pregnant mothers' diversified dietary intake during pregnancy requires improvement.

There is a significant correlation between diversified dietary intake among pregnant mothers and the mother's educational status, meal frequency habits, nutrition knowledge, and knowledge about nutrition during pregnancy [1]. Large numbers of research findings established the relationship between diversified dietary intake and various factors, but the relationship between pregnant mothers' social capital (SC) and their diversified dietary intake have not yet been assessed [4–7].

The concept of social capital is broad and is defined differently by different scholars. According to Lin, SC is a resource embedded in a social structure that is accessed and/or mobilized in purposeful actions [8]. Robert Putnam, consider SC as a feature of social organization characterized by social networks, trust, and norms, which can improve the effectiveness of society through coordinated actions [9]. Other social science scholars describe SC as “a resource that exists in the form of information, ideas, phenomena, and supports obtained through the relationship with other people” [10, 11]. SC is a prominent attribute of Ethiopian communities. Ethiopian social capitals include women's development army, “ider”, which is an informal network of village members or individuals from the same clan or religion that contribute money to support their deceased relatives upon death, “iqube,” which is a traditional saving, religious networks, and neighborhoods. These were established to support each other at times they are in need and also to contribute to economic and societal wellbeing [12, 13].

Given the above significant role of SC in Ethiopian mothers' day-to-day lives, SC might affect pregnant women's dietary intake. In addition, there is evidence of an association between SC and obesity risk reduction, complementary feeding, and self-reported health [14–16], but the association between SC and pregnant mothers' dietary diversity was not studied in Ethiopia. The aim of this study was to determine the proportion of diversified dietary intake and its association with SC among pregnant mothers'.

## 2. Methods

### 2.1. Study Design

A community-based cross-sectional study was conducted in the Arba Minch Zuria district.

### 2.2. Setting

The Arba Minch Zuria district is one of 14 districts in the Gamo Zone, South West of Ethiopia. The study was conducted from February 1 to 29, 2020. The district is geographically situated between 37° 23' 51.37″ Eastern longitude and 5° 55' 16.24″ Northern latitude in the Gamo zone. According to the district administration office report, in June 2019, the district had 128, 743 total population, of which 49.6% were females. Based on South Nation Nationality and People Regional state's (SNNPR) demographic distribution, 3.46% (4455) of the population were pregnant in 2020. Four health centers and 26 health posts have been providing healthcare services to the district people. The area is one of the cash crop growing areas in the SNNPR. Banana, mango, papaya, tomato, lemon, avocado, and maize are commonly produced in the district.

### 2.3. Participants

**Figure 1:**
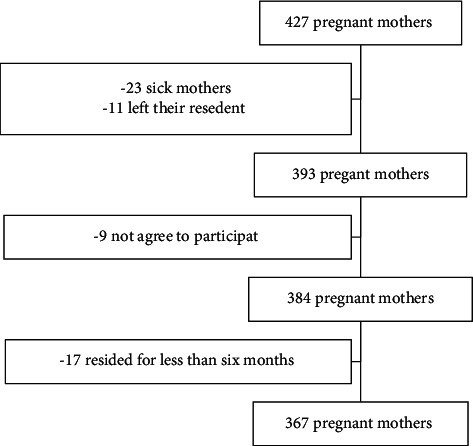
Schematic presentation of the enrollment of participants in the study in Arba Minch Zuria district in February 2021.

Pregnant women living in the Arba Minch Zuria district were the source population. At the time of data collection, pregnant mothers who had lived in the kebeles for at least 6 months were willing to participate in the study, and/or were not ill at the time of data collection took part in the study (Figure 1). Informed consent was obtained verbally from all pregnant mothers.

### 2.4. Variables

The diversified dietary intake of pregnant mothers in the previous 24 hours was the outcome variable. Age, religion, educational status, live with whom, husband's educational status, husband's job, economic status of households, Household Food Insecurity (Access) (HFIA), nutrition during pregnancy knowledge of participants, SC, parity, gravidity (total number of pregnancy), and ANC (World Health Organization defines ANC as, the care provided by educated health-care professionals to pregnant women and adolescent girls in order to ensure the best health conditions for the both mother and baby during pregnancy [17] follow-up were considered as the potential predictors.

### 2.5. Measurement

Data were collected by interview technique using structured and pre-tested questionnaires. The questionnaire was adapted and adopted from different literature [18], and Central Statistical Agency [2, 4, 6, 7, 19–21]. Data were collected using an interview technique using structured and pre-tested questionnaires. The questionnaire was adapted and adopted from different literature. It contains socio-demographic, economic, obstetric conditions, nutritional status, household wealth status, HFI (A), dietary practice, knowledge of nutrition during pregnancy, and dietary intake parts.

Based on FAO and USAID's Food and Nutrition Technical Assistance (FANTA) III Project' recommendation, dietary intake was measured using multiple-pass 24-hour recalls. Its objective was to assess whether the diet of pregnant mothers was adequately diversified or not [19]. According to the recommendations, there are 10 food groups such as grains, white roots and tubers, and plantains, pulses (beans, peas, and lentils), nuts and seeds, dairy, meat, poultry and fish, eggs, dark green leafy vegetables, other fruits and vegetables rich in vitamin A, other vegetables and other fruits. A mother who ate at least five foods from the above lists in the previous 24 hours before the data collection time was considered to be taking adequately diversified food items. A mother who achieved minimum dietary diversity among pregnant mothers is used as a proxy indicator for micronutrient adequacy [22].

The mother's SC was assessed based on the number of social networks in which she participated. Mothers who participated in two or more social networks were labeled as having an SC. Mothers' knowledge about nutrition during pregnancy was assessed using 18 questions about minimum food frequency, diversified dietary intake, anemia, ferrous sulfate with folic acid supplementation, low birth weight, and hygiene. A mother who correctly responded to 14 and more questions (total score > 75%) was considered knowledgeable.

The status of HFI (A) was assessed using the household food insecurity access scale tool adapted from guidelines developed by FANTA [18]. This tool was validated to measure HFI (A) in Ethiopia [23]. The tool consists of nine occurrences and nine frequencies of occurrences questions. There were questions about the occurrence in the form of increased levels of severity of food insecurity. The frequency-of-occurrence question was missed if respondents responded that the condition described in the corresponding occurrence question was not experienced in the previous 30 days. The respondents were also assessed for their economic status indirectly using household factional assets adapted from EDHS 2016 [2].

Mid Upper Arm Circumference (MUAC) was measured using a nonstretchable adult insertion tap to assess nutritional status. The MUAC value of the left arm was taken to the nearest 0.1 cm without clothing on the arm. Pregnant mothers were classified as undernourished when MUAC was <23 cm [24].

### 2.6. Sample Size and Sampling Technique

The sample size was determined using the single population proportion formula. We used a 20.1% proportion of diversified dietary intake from a study conducted in the SNNP region [25] and assumed a 95% confidence level, a 5% margin of error, and a 10% nonresponse rate. The sample size obtained by calculating with the above assumptions was multiplied by the 1.5 design effect since we employed the cluster sampling technique. The sample size then became 407 pregnant mothers. Nine kebeles were selected by a simple random sampling technique and 427 pregnant mothers registered in health posts in selected kebeles were house-to-house assessed with the help of health extension workers for their dietary intakes.

### 2.7. Data Analysis

Dietary intake was analyzed by coding each group of food as “1” or “0” if consumed or not, respectively. The individual responses were then summed into a score ranging from 0 to 10. Each mother was coded as having “adequately diversified dietary intake” if the sum of the score was 5; otherwise, it was coded as “not adequately diversified dietary intake”. HFI (A) was assessed according to the criteria of the FANTA III project and households were classified as food secure, mildly food insecure, moderately food insecure, and severely food insecure [18]. Wealth status was analyzed using the Principal Component Analysis (PCA) technique by considering the presence of fixed and/or productive household assets. First, assets were coded as 0 for the absence of the asset and 1 for the presence of the asset. The variables were then entered and analyzed using PCA, and the variables that had a community value greater than 0.5 were used to produce factor scores. In the end, the index factor scores were classified into three groups: first, second, and third tertiles.

The completed questionnaires were coded and entered into Epi Info™ 7 and analyzed using IBM SPSS Statistics 20 software. Both descriptive and inferential statistical analyses were conducted. In the descriptive analysis, frequency, percentage, mean, and standard deviation were generated and presented using text, tables, and graphs. Inferential analysis was performed in two phases. First, bivariate binary logistic regression was done to identify potential factors that might have an association with diversified dietary intake at the probability value < 0.25. Second, multivariate binary logistic regression was done and the association between independent variables and adequate diversified dietary intake was determined. The factor is considered statistically significant if its AOR 95% CI excludes the null value.

### 2.8. Ethical Considerations

This study was conducted according to the guidelines laid down in the Declaration of Helsinki and all procedures involving research study participants were approved by the Addis Ababa University Institutional Review Board of the College of Natural Sciences (CNSDO/185/12/19). Permission was obtained from the district health office. Written informed consent was obtained from all mothers beside confidentiality was ensured.

## 3. Results

### 3.1. Participants

A total of 427 pregnant mothers registered for the ANC service in nine health posts. Three hundred sixty-seven mothers participated in the study, and the response rate was 90.1%. In terms of nonresponse reasons, 23 were sick, 11 were not available as residents, 17 lived for less than 6 months, and nine refused to provide information.

The average age of the respondents was 25.4 ± 4.8 years. Almost all of them (99.5%) were married, and their median years of marriage were 7 (IQR = 8). There were 4.7 people per household. In terms of nutritional status, 67 (18.3%) of pregnant mothers were undernourished.  Table 1 displays the educational status of mothers and their husbands, as well as the mother's income, household wealth status, and HFI (A).

In terms of obstetric characteristics, 95 (25.9%) mothers were primi gravida, 207 (56%) were multigravida, and 65 (17.7%) were grand multigravida. In terms of gestational age, nearly half of the respondents (179) were in the third trimester, 170 (46.3%) were in the second trimester, and only 18 (4.9%) were in the first trimester. In terms of ANC visits to health facilities, 125 (34%) visited once, 117 (31.9%) visited twice, 82 (22.3%) visited three times, and 43 (11.7%) visited four times.

### 3.2. Diversified Dietary Intake

In the study, the average meal frequency was 3.6 ± 0.9 times per day. According to the survey, 41% of pregnant women had reduced or maintained their food intake frequency compared to their nonpregnant state. Approximately 28% (102) of mothers avoided at least one type of food while pregnant. Among these, 39 percent avoided their main staple foods, “kurkufa,” “fosese,” and “kita” (kurkufa, fosese, and kita are local foods made from maize with cabbage or morringa and/or pulses).  Table 2 shows that many participants were knowledgeable, did not avoid food and fluid during pregnancy, had nutritional information, and supplemented with ferrous sulfate and folic acid.

**Figure 2:**
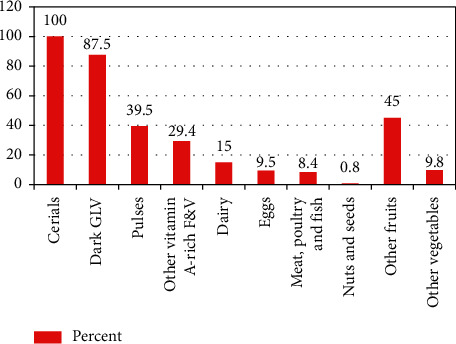
Food groups consumed by pregnant mothers in Arba Minch Zuria district in February 2020.

The study also reveals that Dietary Diversity Score (DDS) was ranged from 2 to 7 and the median DDS was 3 ± 1 SD, which is less than the minimum optimal food groups recommended by FANTA, which is ≥ 5. Only 14.7% (95% CI: 11.1, 18.3) of pregnant mothers took a diversified diet.  Figure 2 shows that all respondents ate starchy staples in the last 24 hours before data collection. Animal-sourced foods, such as meat, fish, poultry, eggs, and dairy products, were on the list of food groups consumed.

### 3.3. Factors Associated with Dietary Intake

Data was used to identify predictors of diversified dietary intake. Mothers' age, education status, years of marriage, income, ANC visit, gravidity, gestational age, husbands' education status, job and emotional support, family size, SC, wealth status, nutrition during pregnancy knowledge, dietary practice, and HFI (A) were identified as potential factors in bivariate analysis and were considered for multivariate binary logistic regression analysis.

Multiple regression analysis revealed that mothers' own income, SC, household wealth status, food frequency, and husband's emotional support were all significantly associated with diversified dietary intake, with AORs of 2.3 (95 percent CI: 1.12, 4.44), 7.8 (95 percent CI: 1.02, 59.19), 2.3 (95 percent CI: 1.04, 5.26), 1.5 (95 percent CI: 1.04, 2.07), and 4.0 (95 percent CI: 1.16, 13.7) (Table 3).

## 4. Discussion

Although SC is a common feature of Ethiopian mothers' lives, its relationship with dietary intake has not been studied. The goal of this study was to determine the proportion of adequately diversified dietary intake and its relationship to SC. We discovered that adequately diversified dietary intake was lower than previously reported and have a statistically significant relationship with SC. To strengthen this finding, a study with a strong design is required.

### 4.1. Diversified Dietary Intake

Our study's proportion of adequately diverse dietary intake was lower than figures from South Africa, Kenya, Ghana, and Pakistan, which were 25 percent, 61 percent, 46 percent, and 94 percent, respectively [26–29]. Similarly, the finding of this study was lower than the 41 percent obtained by a systematic review of studies conducted in Ethiopia, and the 24.7 percent obtained by another community-based study conducted in the Gamo zone, Kucha district [1, 30]. This is because the study was conducted in a rural area, where mothers may have limited access to information and may adhere to traditional dietary intake practices.

### 4.2. Factors Associated with Adequate Dietary Intake

This study found no statistically significant link between adequate dietary diversity intake and certain demographic factors (mothers' age, religion, husband education, and husband occupation). This finding is similar to one based on an Ethiopian systematic review [1]. However, mothers who earn their own money have a better chance of eating a diverse diet than mothers who do not. Similar to this study, other studies that looked at the effect of income, using average monthly income, on dietary intake found that pregnant mothers from households with the highest monthly income had adequate dietary practices more frequently than their counterparts [21, 28, 31, 32]. This is because income goes with decision-making power, as it may increase the ability to purchase a variety of food groups.

SC was a significant factor in determining adequate dietary intake. Mothers who had SC were nearly eight times more likely to have an adequate diversified diet (AOR: 7.8; 95 percent CI: 1.02, 59.19) than mothers who did not benefit. There was no evidence of a statistical association between SC and a diverse dietary intake. However, evidence from rural Ethiopia suggests that mothers with SC were more than twice as likely as nonmembers to achieve their child's DDS [15]. Our findings provide empirical evidence for a link between dietary intake and SC.

In terms of food frequency habits, the findings show that increasing meal frequency is significantly associated with a diverse dietary intake. When a mother increased her food frequency by one, her chances of achieving an adequately diverse dietary intake increased by more than one (AOR: 1.5, 95 percent CI: 1.04, 2.07). Our findings are comparable to those of Jemal and Awol's study at Alamata General Hospital [33].

Based on the wealth status of the household, mothers in the third tertile household had more than twice the odds of taking an adequately diversified diet (AOR = 2.3, 95% CI: 1.04, 5.26) compared to mothers of the first tertile household. Other previous studies have found a consistent relationship between wealth status and adequate dietary diversity practice [1, 4, 27, 34]. A study conducted in Northeast Ethiopia, for example, found that a mother from a third tertile household was nearly twice as likely as a mother from a first tertile household to achieve adequate dietary intake [4]. This could be because mothers from third-tertile households have better access to and capacity to purchase diverse foods.

Another statistically significant factor that affects adequate dietary intake was the emotional support of the husband. A pregnant mother who had a husband's emotional support during the time of pregnancy was four times more likely (AOR: 4.0, 95% CI: 1.16, 13.7) to have an adequately diversified diet than a mother who did not have a husband's emotional support. This is because, in Ethiopia, many houses are patriarchal in nature and the husband is the most decisive person in most household issues; if the husband was involved in pregnancy issues, pregnant mothers were likely to obtain adequately diversified food. The finding in central Ethiopia is also similar to our finding. As a result, mothers who received emotional support from their husbands are more likely to consume a diverse diet than mothers who did not receive emotional support from their husbands [6].

A considerable body of evidence asserts the relationship of SC with complementary feeding, self-reported health, and obesity reduction. We found an association between SC and diversified dietary intake. Hence, incorporating SC as a nutrition intervention might help more to enhance pregnant mothers' dietary intake.

### 4.3. Limitation and Strength of the Study

The study based in a community setting would improve the precision of estimating the dietary intake of mothers, unlike an institution-based study. Although a multiple-pass 24-hour recall is used to reduce recall bias, a single 24-hour recall is not as strong to describe mothers' usual dietary intake because of the possibility of day-to-day or intra-individual variability.

## 5. Conclusion

This study showed that adequate dietary intake was low. Mothers with SC were benefited by enhancing their dietary intake. However, more studies are required based on a strong study design to further strengthen the empirical evidence on the effect of SC on dietary intake.

## Figures and Tables

**Table 1:** Demographic and socioeconomic characteristics of pregnant mothers in South West Ethiopia in February 2020 (*n* = 367).

Characteristics	Categories	Number	Percent
Educational status	Illiterate	66	18
	Attended some grads	266	72.5
	Higher education	35	9.5

Living	With husband	344	93.7
	Alone	2	0.5
	Her family	9	2.5
	Husband family	12	3.3

Religion	Protestant	297	80.7
	Orthodox tewahedo	68	18.5
	Muslim	2	0.5

Mothers have income	Yes	90	24.5
	No	272	75.5

Husband's education status	Illiterate	54	14.7
	Attend some grades	269	73.3
	Higher education	44	12

Husband's job	Government office	37	10.1
	Farmer	145	39.5
	Retailor	85	23.2
	Daily wage	100	27.2

Husband's support	Yes	301	82
	No	66	18

Participating in social network	Yes	312	85
	No	55	15

Wealth status	Poor	122	33.2
	Medium	125	34.1
	Rich	120	32.7

HFI (A)	Food secured	230	62.7
	Mild food insecure	34	9.30
	Moderately food insecure	67	18.3
	Severely food insecure	36	9.8

HFI (A): household food insecurity access.

**Table 2:** Nutritional practice of pregnant mothers in South West-Ethiopia, in February 2020 (*n* = 367).

Characteristics	Categories	Number	Percent
Knowledge of nutrition during pregnancy knowledge	Knowledgeable	308	83.9
	Not knowledgeable	59	16.1

Avoid any food or fluid	Yes	102	27.8
	No	265	72.2

Nutrition information	Yes	201	45.2
	No	166	54.8

Iron tablet intake status	Taking	249	67.8
	Stop taking	46	12.5
	New	72	19.6

**Table 3:** Factors associated with dietary diversity practice of pregnant mothers in Arba Minch Zuria district, South West-Ethiopia, February 2020 (*n* = 367).

Variable	Category	Dietary practice		COR (95% CI)	AOR (95% CI)
		Inadequate	Adequate		
Age		313	54	1.1 (1.0, 1.1)	

Mother's education	Illiterate	55 (83.3%)	11 (16.7%)	1	
	Some grade attended	234 (88%)	32 (12%)	0.7 (0.3, 1.4)	
	Higher education	24 (68.6%)	11 (31.4%)	2.3 (0.9, 6.0)	
Years in marriage		313	54	1.1 (1.0, 1.1)	

Mother having income	No	243 (89.5%)	29 (10.5%)	1	1
	Yes	65 (72.2%)	23 (27.8%)	3.3 (1.8, 6.0)	2.3 (1.1, 4.4)

Husband's education	Illiterate	46 (85.2%)	8 (14.8%)	1	
	Some grade attended	235 (87.4%)	34 (12.6%)	0.8 (0.4, 1.9)	
	Higher education	32 (72.7%)	12 (27.3%)	2.2 (0.8, 5.9)	

Husband's job	Government employee	28 (75.7%)	9 (24.3%)	7.7 (2.2, 26.9)	
	Farmer	118 (81.4%)	27 (18.6%)	5.5 (1.9, 16.2)	
	Retailer	71 (83.5%)	14 (16.5%)	4.7 (1.5, 15.0)	
	Daily labor	96 (96%)	4 (4%)	1	
ANC visit		313	54	1.3 (0.96, 1.6)	

Gravida	One	87 (91.6%)	8 (8.4%)	1	
	Two up to five	178 (86%)	29 (14%)	1.8 (0.8, 4.0)	
	More than five	48 (73.8%)	17 (26.2%)	3.9 (1.5, 9.6)	

Gestational age	First trimester	14 (77.8%)	4 (22.2%)	1	
	Second trimester	148 (87.1%)	22 (12.9%)	0.5 (0.2, 1.7)	
	Third trimester	151 (84.4%)	28 (15.6%)	0.6 (0.2, 2.1)	
Family size		313 (4; IQR : 3)	54 (5; IQR : 2)	1.1 (1.0, 1.3)	

SC	No	53 (98.1%)	2 (1.9%)	1	1
	Yes	260 (83.1%)	52 (16.9%)	10.8 (1.5, 79.9)	7.8 (1.0, 59.2)

Wealth	First tertile	112 (91.8%)	10 (8.2%)	1	1
	Second tertile	114 (91.2%)	11 (8.8%)	1.1 (0.4, 2.6)	0.8 (0.3, 1.9)
	Third tertile	87 (72.5%)	23 (27.5%)	4.2 (2.0, 9.1)	2.3 (1.1, 5.3)

Knowledge of nutrition during pregnancy knowledge	Not knowledgeable	266 (86.4%)	42 (13.6%)	1	
	Knowledgeable	47 (79.7%)	12 (20.3%)	1.6 (0.8, 3.3)	
Frequency of eating		313	54	1.6 (1.2, 2.2)	1.5 (1.1, 2.1)

Husband's support	No	61 (95.3%)	5 (4.7%)	1	1
	Yes	252 (83.2%)	49 (16.8%)	4.1 (1.2, 13.6)	4.0 (1.2, 13.7)

HFIA¥	Secured	188 (81.7%)	42 (18.3%)	2.5 (0.7, 8.4)	
	Mild insecure	29 (85.3%)	5 (14.7%)	1.9 (0.4, 8.6)	
	Moderately insecure	63 (94%)	4 (6%)	0.7 (0.1, 3.3)	
	Severely insecure	33 (91.7%)	3 (8.3%)	1	

## Data Availability

The data that support the findings of this study are available on request to the corresponding author. The data are not publicly available due to privacy or ethical restrictions.

## Abbreviations

AOR: Adjusted odds ratio
EDHS: Ethiopian demographic health survey
FANTA: Food and nutrition technical assistance
FAO: Food and agriculture organization of the United States
IQR: Interquartile range
HFI (A): Household food insecurity access
MDDW: Minimum dietary diversity for women
MUAC: Mid upper arm circumference
PCA: Principal component analysis
SNNPR: South Nation Nationalities Regional State
SPSS: Statistical Package for Social Sciences
SD: Standard deviation.
